# Aerosol Test Chambers: Current State and Practice During the COVID-19 Pandemic

**DOI:** 10.3389/fbioe.2022.863954

**Published:** 2022-04-12

**Authors:** Kenneth B. Yeh, Bradly Setser

**Affiliations:** ^1^ MRIGlobal, Kansas City, MO, United States; ^2^ JRAD, Stafford, VA, United States

**Keywords:** aerobiology, aerosol test chamber, bioaerosol, biodefense, biosecurity

## Abstract

Respiratory infectious disease outbreaks such as those caused by coronaviruses and influenza, necessitate the use of specialized aerosol test chambers to study aspects of these causative agents including detection, efficacy of countermeasures, and aerosol survivability. The anthrax attacks from 2001 and earlier biowarfare and biodefense also influenced the study of biological aerosols to learn about how certain pathogens transmit either naturally or through artificial means. Some high containment biological laboratories, which work with Risk Group 3 and 4 agents in biosafety level -3, biosafety level-4 containment, are equipped with aerosol test chambers to enable the study of high-risk organisms in aerosolized form. Consequently, the biomedical, military and environmental sectors have specific applications when studying bioaerosols which may overlap while being different. There are countless aerosol test chambers worldwide and this number along with numerous high containment biological laboratories underscores the need for technical standards, regulatory and dual-use compliance. Here we survey common aerosol test chambers and their history, current use, and practice. Our findings reinforce the importance and need for continued collaboration among the multi-disciplinary fields studying aerobiology and biological aerosols.

## Background

Aerosol test chambers (ATC) are specialized facilities used to house studies for detection, animal models, and related efforts involving dispersal of biological aerosols (bioaerosols). ATCs are common among academic, commercial, government, and non-profit research organizations where they often employ researchers who perform studies involving these facilities and maintaining such equipment. For example, ATCs are used to study aerosols containing biological material and minimize the physical loss of particles to isolate key variables and study specific aerosol parameters over time or to infect animal subjects to a relevant dose (Santarpia et al., 2020). These facilities also have essential equipment such as biosafety cabinets (BSC), nebulizers or other dissemination mechanisms, fans, particle sizers, and sample collectors used to study bioaerosol particle dynamics across multiple disciplines including infectious disease, environmental pollution studies, industrial hygiene, and biodefense (Ho et al., 2001; Smither et al., 2020; Lackemeyer et al., 2014). ATCs may vary in size from benchtop-sized boxes that can range in size from 1 to 2 cubic meters to rooms that vary in size from approximately 30–50 cubic meters. ATCs are also classified according to their appropriate biosafety level (BSL-1, BSL-2, BSL-3, BSL-4) and/or corresponding animal biosafety level (ABSL-3, or ASBL-4). These classifications are based on requirements for primary containment such as BSCs, secondary containment (physical infrastructure), and other engineering controls. The biosafety levels and specific requirements described depend on the given risk-based assessment which is part of biosafety best practice conducted prior to initiating work. This assessment also takes into account the risk group of any biological agent that is permitted to be aerosolized within the ATC.

Although the World Health Organization designates SARS-CoV-2 as a Risk Group 3 biological agent, it can be handled at the BSL-2 level for routine diagnostic analysis (ABSA, Kaufer et al., 2020). However, BSL-3 level containment is often required for activities such as culturing high concentrations of virus and handling highly transmissible variants. BSL-3 containment provides negative pressure airflow to ensure that aerosols do not escape the laboratory, requires individual respiratory protection, and provides an area for donning and doffing of personal protection equipment (Centers for Disease Control and Prevention, 2021; Yeh et al., 2021). These containment features can help prevent zoonosis from laboratory animals and potential exposure to aerosols as could be the case using ATCs. The essential equipment used in an ATC for dissemination, measurement, and collection all factor into a risk-based assessment which are often developed on a case-by-case basis.

National state programs became active in biodefense in the first half of the 20th century, as consequence of World War II (WWII), and during the Cold War. The Geneva and Biological Weapons Conventions banned offensive use of biowarfare agents (BWA) thus reinforcing biodefense measures for studying detection, survivability and dispersal. Biodefense studies involving BWA may include actual pathogens or simulants and dispersals occurring indoors such as aerosol chamber and outdoors such as field test (Manchee et al., 1981). The Former Soviet Union, which ran an offensive biowarfare program until the early 1990s, also had large and small ATCs and equipment to make BWA weapons (GAO, 2000). Performing indoor studies using ATCs is more suitable and practical due to the ability to control environmental variables such as temperature, relative humidity, wind speed, and particle concentration. These variables are more difficult to manage especially large open air challenges held in the field outdoors. Certain sites such as the Dugway Proving Ground (Utah, United States), Gruinard Island (United Kingdom), and the Defence Research Establishment Suffield (Medicine Hat, Canada) developed unique biodefense capabilities for indoor aerosol chamber and outdoor field testing (Manchee et al., 1981; Ho et al., 2001; Beedham and Davies, 2021). Collaborations among US, United Kingdom, Canada, and others continue today.

In general, aerosol work would be difficult to detect, monitor, and verify. This is especially true for indoor aerosol studies using ATCs where it is hard to discern dual-use equipment. Aerobiology study methods are often straight-forward but descriptions for equipment set up, agent dispersion, sampling, collection, and detection are lengthy due to the inherent physical and biological nature. Frameworks such as the Australia Group were established to maintain an export control list of dual-use equipment (Australia Group, 2022). Tracking such equipment through export controls also counters the spread of illicit use of technology to propagate BWA.

Although there is no standard biosafety oversight, WHO and CDC provide widely accepted biosafety guidelines which aid at the national level (World Health Organization, 2020; Meechan and Potts, 2020). WHO and NIH provide guidance for dual-use research of concern (Stavskiy et al., 2003; National Academies Science Engineering Medicine, 2017). Related references especially for high containment applications can be found in the literature. Although ATCs are not specifically called out in the referenced biosafety guidance, they are often associated and featured in high containment- BSL-3 and BSL-4 work due to the nature of risk group pathogens and related exposure on a case basis according to specific risk assessment (ABSA 2021). Although pathogens such as anthrax and SARS-CoV-2 are zoonotic in nature, research continues to be heavily scrutinized to prevent unintended consequences and misuse (if not attribution) where there is risk for gain-of-function or unintended release.

## Measuring Bioaerosols-Still Very Much Art and Science

In addition to bioaerosol research aspects and dual-use applications, recent publications also address the need for standardized methods, harmonized regulations, and increased multi-disciplinary initiatives (Mubareka et al., 2019; Mainelis, 2020). Although there are no authoritative standards that directly address the use of ATCs, there are numerous standards that address other aerosol-specific disciplines from a product and safety standpoint. For example, the International Organization for Standardization (ISO) 20,072:2009 applies to the design, labeling, instruction for use and testing requirements for hand-held single- and multi-use aerosol drug delivery device and the National Institute for Occupational Safety and Health provides analytical methods for industrial hygiene monitoring (Lindsley et al., 2017; ISO, 2021). It is especially difficult to derive a unit of measurement for health risk that incorporates factors for physical and biological properties on an aerosolized agent and susceptible population (National Research Council, 2008). Traditionally, bioaerosol units of measure depend on mechanisms of detection, identification, and the type of agent dispersed whether it is bacteria, fungal, viral. While this approach is useful in understanding the overall aerosol concentration as it pertains to the performance of a detector or identifier or other type of equipment being evaluated (i.e., protection offered by a mask), it is does not fully characterize the health risk.

Generally, an aerosol particle per volume (liter or cubic meter of air) does not indicate that particle contains the target agent. A simple particle counter like an Aerodynamic Particle Sizer® would measure particle per liter (P/L) of air. For culturing bacteria and viruses, biologists commonly use colony forming unit (CFU) and plaque forming unit (pfu) which can also be used to measure bacteria (i.e. CFU/liter of air) or viruses (i.e., pfu/liter of air) in the air from liquid or solid collection. The US Department of Defense (DOD) has historically used the term Agent Containing Particles of liter of air (ACPLA) to describe a particle that has a detected target organism within it; to differentiate it from simply a particle per liter of air which provides comparable data when evaluating detectors but little operational utility (National Research Council, 2008; Valdes et al., 2010). Furthermore, with the advent of detection and identification systems based on DNA amplification (i.e., polymerase chain reaction (PCR), DOD and US Department of Homeland Security have also used genomic equivalents (GE) per volume of air, however this measure assumes that each organism or virion has one genome or copy of DNA, and does not account for plasmids or free-floating DNA. The DOD and stakeholders convened where they proposed a new unit: Biologically Active Units per Liter of Air as a function of aerodynamic diameter (BAULADae) (National Research Council, 2008). Another evaluation proposed another unit of measurement called Total Agent per Liter of Air with particle size distribution (TALAp) to address two most important variables for evaluating biodetectors: the amount of agent present and the particle size distribution (Valdes et al., 2010). It is likely the unit of measurement will be considered on a case-by-case basis as this also depends on many variables including the equipment used to disperse and measure the bioaerosol.

## Basic Types of Aerosol Test Chambers

Due to their broad and multi-disciplinary use, aerosol chambers generally exist as one of three categories, Static Chambers, Flow-Through Chambers, and Rotating Chambers (Santarpia et al., 2020). Static and Rotating Chambers have basic and conventional designs that reflect their long use over several decades since the WWII era. Although developed later, Flow-Through Chambers, which have incorporated additional controls and filtering, are also widely used. Static Chambers are those constructed so that no additional air is introduced beyond what was present at the beginning of the experiment. Fans fixed inside the Static Chambers can facilitate mixing of aerosolized material and counteract gravitational settling. They are the least complex of the three categories of chambers and their primary limitation is the relatively rapid loss of aerosol due to gravity over time.

Flow-Through Chambers, also referred to as Dynamic or Wind-Tunnel Chambers, are more complex and incorporate air that is continuously introduced into the chamber using airflow velocity to counteract the loss of aerosol due to gravity over the course of an experiment. This enables researchers and testers to mimic a realistic airflow environment, however they require continuous aerosol dissemination to maintain aerosol concentration over time. Rotating Chambers, which are also referred to as Goldberg Drum or Rotating Drum Chambers, are typically constructed as a cylinder that rotates over time to reduce the effects of gravity and to maintain aerosol aloft for longer time when compared to a static chamber (Goldberg et al., 1958). The rotating drum has been used extensively since it was first developed and has been referred to as “probably the standard procedure used for aerosol longevity studies” (Haddrell and Thomas, 2017; Verreault et al., 2013; May and Druett, 1968). To address these limitations, the rotating drum methodology has been enhanced over time including enabling its use in containment (Hommema et al., 2014), improved environmental controls (i.e., relative humidity, temperature and ultraviolet radiation) (Fischer et al., 2016; Pan et al., 2014; Ratnesar-Shumate et al., 2015; Verrault et al., 2014), and testing outdoors with transparent and gas permeable membrane exterior wall (Santarpia et al., 2017). While the methods employing the use of these ATCs are established, the lack of standardization reflects the current state of the art regarding the use of ATCs for biodefense purposes.

## Examples of Related Aerosol Test Chamber Work

As mentioned previously, biodefense programs in the US, United Kingdom, and Canada have continued bioaerosol work domestically and jointly since WWII. In the US, academic, industry, and Government organizations with ATC capabilities have performed research and related activities in their respective niche market that have traditionally experienced low demand. Some of these markets included industrial hygiene applications such as monitoring exposure to environmental chemicals and molds. Nonprofit research organizations have also historically provided related biotech research services for translational sciences including diagnostics, therapeutics, and vaccines (Moos and Mirsalis, 2009). The convergence of biotech and biopharma with respiratory infectious diseases has resulted in additional demand for bioaerosol services that military and biodefense performed. More recent real world examples include countering weapons of mass destruction and terrorism involving BWAs and infectious disease outbreaks:

• Suspected use of chemical and biological weapons during the two Gulf Wars in 1991 and 2003.
• The terrorist attacks on New York City and Washington DC on 11 September 2001.
• The 2001 *B. anthracis* attacks in the United States in October 2001.
• Outbreaks of SARS, MERS-CoV, ebolaviruses in 2000s.
• Outbreak of SARS-CoV-2 in 2019 and COVID-19 pandemic.

There are numerous aerosol test chambers worldwide represented across academia, industry and government. A few examples of each category are shown (Table 1). For the COVID-19 pandemic specifically, several entities have responded by utilizing existing infrastructure, or repurposing infrastructure and/or expertise to study the virus. For example, prior to the pandemic, Tulane University utilized a rotating drum chamber to study the long-term effects of environmental conditions on airborne organisms (Verreault et al., 2014]. During the pandemic, Tulane, participating in a study with the University of Pittsburgh, National Institutes of Health-Integrated Research Facility, and US Army Medical Research Institute of Infectious Diseases, repurposed their aerosol expertise to study the effects of Severe Acute Respiratory Syndrome (SARS) on primates using a head-only exposure chamber (Fears et al., 2020). MRIGlobal which has an ATC in a BSL-3 also supported various commercial studies for their respective COVID-19 products (Yeh et al., 2021).

**TABLE 1:** Representative set of aerosol test chambers. The list was derived from open source material.

Organization		Type	Biosafety level
Academia	Tulane University	Flowthrough	ABSL-3
	University of Pittsburgh	Flowthrough	BSL/ABSL-3
Commercial	CUBRC-Avarint	Static, Flowthrough	BSL-1, 2
	Battelle	Flowthrough	BSL-3
	Lovelace Respiratory Research Institute (LRRI)	Flowthrough	BSL-3/ABSL-3
	MRIGlobal	Static, Flowthrough	BSL-3
Government	Dugway Proving Ground	Static, Flowthrough	BSL-3
	National Biodefense Analysis and Countermeasures Center (NBACC)	Static, Flowthrough, Rotating	BSL-3
	U.S. Army Medical Institute for Infectious Diseases (USAMRIID)	Static Flowthrough	BSL/ABSL4
	Defence Science and Technology Laboratory (Dstl)	Flowthrough, Rotating	BSL-4

Another example of an entity repurposing their expertise to study SARS-CoV-2 is the US Army Dugway Proving Ground (DPG). In addition to biodefense, DPG provides testing and support for counter CBRE hazards (U.S. Army Dugway Proving Ground, 2022), and they have a broad range of bioaerosol chambers to test military hardware against biological warfare threats. Following the COVID-19 outbreak, these assets focused on DPG’s ongoing mission to test military hardware against biological warfare agents. However, DPG aerobiologists and biological agent experts have initiated a study on a commercial airplane fuselage using a simulant for SARS-CoV-2 to gauge the effectiveness of two commercial decontaminants and sprayers (Military Health System, 2022).

Similar to the United States, foreign governments have entities that focus on infectious disease, to include BWA. An allied counterpart in the United Kingdom is their Defence Science and Technology Laboratory (Dstl) which the Ministry of Defense operates at Porton Down. Prior to the COVID 19 outbreak, Dstl conducted numerous studies using a Rotating Drum to study the stability and viability of aerosolized infectious diseases, including Lake Victoria Marburgvirus, Zaire ebolavirus, and Reston ebolavirus (Piercy et al., 2010; Schuit et al., 2014; Beedham et al., 2021). Following the COVID 19 outbreak, Dstl used this same capability to study SARS-CoV-2 in saliva and different media at various humidity (Smither et al., 2020).

## Conclusion

Recent descriptions of high containment biological laboratories that demonstrated their utility and lessons learned during COVID-19 pandemic offer unique capability that has become essential during the recent outbreaks of respiratory infectious disease (Yeh et al., 2021). Besides laboratory diagnostics, these experiences are also relevant to ATCs where research on decontamination, therapeutics, vaccines, along with related bioaerosol testing had to be prioritized during the pandemic. It is difficult to translate these results to inform public health and mitigate risks from respiratory pathogens in a real-world environment. The highly infectious nature of SARS-CoV-2 virus and the demand for using ATCs has increased during COVID-19 similar to the period after the 2001 anthrax letter attacks when the interest in *B. anthracis* and bioterrorism was high. However, ATCs and related bioaerosol efforts have historically been a niche and service-oriented market resulting in a lack of standardized methods for ATC use and measuring bioaerosols.

Often the use of ATCs does not accommodate the different kinds of research being conducted. As described, the basic types of ATCs are inherently inflexible. For example, if a researcher is studying infection pathways or infectious dose, they are primarily limited to ABSL Flowthrough chambers, or if studying aerosol survivability in the environment, researchers will primarily use Rotating Chambers or Static Chambers. Another consideration is the artificiality inherent in using ATCs such as the inability to replicate the relevant real-world environment including UV radiation and pollutants. Researchers have attempted to address this artificiality over time by enhancing capabilities, but it is important that researchers consider these parameters when analyzing results.

While the number of HCBLs continues to increase for many reasons as countries and states will choose to prioritize and build them, the number of ATCs will likely increase since the small, portable facilities have lower startup cost. In addition, ATCs are often not under governmental oversight, which make it difficult to estimate their exact numbers. Further work is needed to define methods according to perspectives of health-risk to humans, product-performance of detectors and efficacy of treatments. While challenging, the multi-disciplinary nature of the work offers potential opportunities for greater collaboration among respective communities, which encourages greater transparency through trust building.

Responsible development of bioaerosol capabilities should also be considered in the future as related to maintaining resilient biotech industrial base. As demonstrated in the COVID-19 pandemic, the so-called “warm-base” model is essential for preparedness for supplementing manufacture of disposable consumables such as swabs and personal protective equipment. In addition, biomanufacturing of commonly used reagents such as viral transport medium and vaccines require specific materials and scale-up process which are difficult and expensive to have on-demand. These capabilities and associated markets support the bioeconomy ecosystem as a whole.

## Data Availability

The original contributions presented in the study are included in the article/Supplementary Material, further inquiries can be directed to the corresponding author.
