# Corrigendum: Aerosol test chambers: Current state and practice during the COVID-19 pandemic

**DOI:** 10.3389/fbioe.2022.1036662

**Published:** 2022-10-20

**Authors:** Kenneth B. Yeh, Bradly Setser

**Affiliations:** ^1^ MRIGlobal, Kansas City, MO, United States; ^2^ JRAD, Stafford, VA, United States

**Keywords:** aerobiology, aerosol test chamber, bioaerosol, biodefense, biosecurity

In the published article, there was an error in Table 1 as published. The government organization “Defence Science and Technology Laboratory (Dstl)” should be listed as having both Flowthrough and Rotating types at Biosafety level (BSL)-4 instead of BSL-3. The correct Table 1 appears below. The original article has been updated.

**TABLE 1:** Representative set of aerosol test chambers. The list was derived from open source material.

Organization		Type	Biosafety level
Academia	Tulane University	Flowthrough	ABSL-3
	University of Pittsburgh	Flowthrough	BSL/ABSL-3
Commercial	CUBRC-Avarint	Static, Flowthrough	BSL-1, 2
	Battelle	Flowthrough	BSL-3
	Lovelace Respiratory Research Institute (LRRI)	Flowthrough	BSL-3/ABSL-3
	MRIGlobal	Static, Flowthrough	BSL-3
Government	Dugway Proving Ground	Static, Flowthrough	BSL-3
	National Biodefense Analysis and Countermeasures Center (NBACC)	Static, Flowthrough, Rotating	BSL-3
	U.S. Army Medical Institute for Infectious Diseases (USAMRIID)	Static Flowthrough	BSL/ABSL4
	Defence Science and Technology Laboratory (Dstl)	Flowthrough, Rotating	BSL-4

In the published article, the reference for Beedham and Davies, 2021 was inadvertently included in the last sentence of the last paragraph in the section, “Examples of Related Aerosol Test Chamber Work”. It should be added to the preceding sentence of the same paragraph in the same section.

The sentences previously stated:

“Similar to the United States, foreign governments have entities that focus on infectious disease, to include BWA. An allied counterpart in the United Kingdom is their Defence Science and Technology Laboratory (Dstl) which the Ministry of Defense operates at Porton Down. Prior to the COVID 19 outbreak, Dstl conducted numerous studies using a Rotating Drum to study the stability and viability of aerosolized infectious diseases, including Lake Victoria Marburgvirus, Zaire ebolavirus, and Reston ebolavirus (Piercy et al., 2010, Schuit et al., 2014). Following the COVID 19 outbreak, Dstl used this same capability to study SARS-CoV-2 in saliva and different media at various humidity (Smither et al., 2020; Beedham and Davies, 2021).”

The corrected sentences appear below:

“Similar to the United States, foreign governments have entities that focus on infectious disease, to include BWA. An allied counterpart in the United Kingdom is their Defence Science and Technology Laboratory (Dstl) which the Ministry of Defense operates at Porton Down. Prior to the COVID 19 outbreak, Dstl conducted numerous studies using a Rotating Drum to study the stability and viability of aerosolized infectious diseases, including Lake Victoria Marburgvirus, Zaire ebolavirus, and Reston ebolavirus (Piercy et al., 2010; Schuit et al., 2014; Beedham and Davies, 2021). Following the COVID 19 outbreak, Dstl used this same capability to study SARS-CoV-2 in saliva and different media at various humidity (Smither et al., 2020).”

The authors apologize for this error and state that this does not change the scientific conclusions of the article in any way. The original article has been updated.
